# Predicting Individual Pain Thresholds From Morphological Connectivity Using Structural MRI: A Multivariate Analysis Study

**DOI:** 10.3389/fnins.2021.615944

**Published:** 2021-02-10

**Authors:** Rushi Zou, Linling Li, Li Zhang, Gan Huang, Zhen Liang, Zhiguo Zhang

**Affiliations:** ^1^School of Biomedical Engineering, Health Science Center, Shenzhen University, Shenzhen, China; ^2^Guangdong Provincial Key Laboratory of Biomedical Measurements and Ultrasound Imaging, Shenzhen University, Shenzhen, China; ^3^Marshall Laboratory of Biomedical Engineering, Shenzhen University, Shenzhen, China; ^4^Peng Cheng Laboratory, Shenzhen, China

**Keywords:** pain sensitivity, structural MRI, morphological connectivity, individual difference, multivariate analysis

## Abstract

Pain sensitivity is highly variable among individuals, and it is clinically important to predict an individual’s pain sensitivity for individualized diagnosis and management of pain. Literature has shown that pain sensitivity is associated with regional structural features of the brain, but it remains unclear whether pain sensitivity is also related to structural brain connectivity. In the present study, we investigated the relationship between pain thresholds and morphological connectivity (MC) inferred from structural MRI based on data of 221 healthy participants. We found that MC was highly predictive of an individual’s pain thresholds and, importantly, it had a better prediction performance than regional structural features. We also identified a number of most predictive MC features and confirmed the crucial role of the prefrontal cortex in the determination of pain sensitivity. These results suggest the potential of using structural MRI-based MC to predict an individual’s pain sensitivity in clinical settings, and hence this study has important implications for diagnosis and treatment of pain.

## Introduction

Pain is a multidimensional subjective experience, which exhibits huge inter-individual variability (Rainville, 2002; Coghill, 2010). Pain experience is largely determined by an individual’s sensitivity to pain, which is highly variable across individuals (Nielsen et al., 2009). Investigating the underlying mechanism of individual difference in pain sensitivity has attracted an ever-increasing interest, because it can reveal the individual traits of pain sensitivity and will further enable developing predictive models of individual pain sensitivity. This stream of research has great medical implications both for healthcare systems (Schulz et al., 2012) and for pharmaceutical research (Chizh et al., 2009). For instance, accurate prediction of pain sensitivity could reduce the rate of postsurgical clinical pain (Werner et al., 2010; Abrishami et al., 2011).

Individual differences in pain sensitivity could be attributed to many factors, from genetics to sociocultural variables. Because pain is processed and perceived in the brain, neural mechanism of pain sensitivity is generally recognized as playing a major role in the representation and modulation of pain (Rainville, 2002; Apkarian et al., 2005). By using neuroimaging approaches such as magnetic resonance imaging (MRI) and electroencephalography (EEG), many studies have succeeded in linking the individual pain sensitivity to the variability in brain structure and function. Functional MRI (fMRI) studies indicated that the pain-induced blood oxygen level-dependent (BOLD) responses of the primary somatosensory cortex (SI), anterior cingulate cortex (ACC), and prefrontal cortex (PFC) are positively related to individual pain sensitivity (Coghill et al., 2003), while some regions within the posterior parietal cortex also contribute to individual differences in pain sensitivity by directing attention to painful stimuli (Oshiro et al., 2007; Lobanov et al., 2013). Structural MRI studies have shown that the differences in gray matter (GM) and cortical thickness of the brain are correlated with the individual differences in pain perception in healthy subjects. Emerson et al. (2014) found that GM intensity in bilateral regions of the posterior cingulate cortex, precuneus, intraparietal sulcus, and inferior parietal lobule and in unilateral regions of the left SI showed a significant inverse relationship with pain sensitivity. Another GM analysis also found a strong correlation between pain sensitivity and cortical thickness of the SI cortex (Erpelding et al., 2012). A voxel-based morphometry study (Ruscheweyh et al., 2018) revealed that Pain Sensitivity Questionnaire scores, which assessed the pain ratings of imagined painful situations, were positively correlated with GM volume (GMV) of the parahippocampal gyrus. In summary, these studies have accumulated abundant evidence of multimodal MRI-based neural correlates of individual pain sensitivity.

Most of the existing neuroimaging studies of pain sensitivity focused on the relationship between the individual pain sensitivity and the structure or function of specific brain regions. However, pain is a complex experience related to a wide network of brain regions. Hence, investigating the association between pain sensitivity and brain connectivity can provide a new sight into the neural basis of pain sensitivity. A variety of brain connectivity can be estimated from multimodal MRI data. For example, functional connectivity is normally estimated from fMRI as the statistical relationship between fMRI signals of different brain regions, while structural connectivity can be inferred from T1 images and diffusion tensor imaging (DTI). Several studies have demonstrated that functional connectivity between some specific regions is related to pain perception and can be used as a neural indicator of individual pain sensitivity. Tu et al. (2019) used multivariate pattern analysis to find that resting-state functional connectivity could be used to predict individual pain threshold with high accuracy (a correlation coefficient of 0.60 between predicted and real values of heat pain thresholds). Spisak et al. (2020) identified and validated a pain-free resting-state functional connectivity pattern that is predictive of individual differences in pain sensitivity. On the other hand, structural connectivity can be constructed using mainly two approaches: tractography for DTI and structural covariance network analysis for T1-weighted images. By using the graph analysis of probabilistic tractography based on DTI, one study found that the anterior insula connectivity was related to the individual degree of pain vigilance and awareness (Wiech et al., 2014). As for T1-weighted images, morphological connectivity (MC) can be inferred by calculating the inter-regional similarities of local brain morphology (Tijms et al., 2012; Batalle et al., 2013; Kong et al., 2015). Although MC has been shown to be important neural markers of perception (Li and Kong, 2017) and neurological diseases (Wang et al., 2020), it has not been used to study individual pain sensitivity. As compared with fMRI and DTI, T1-weighted MRI has distinct advantages in its easy access, high signal-to-noise ratio, and relative insensitivity to artifacts (e.g., head motion). Thus, MC inherits the advantages of T1-weighted MRI and is promising to serve as another canonical tool in characterizing structural connectivity related to individual pain sensitivity (Alexander-Bloch et al., 2013a; Evans, 2013). There is mounting evidence suggesting the potential use of MC in predicting individual pain sensitivity. First, the brain’s morphological information is important in shaping an individual’s pain experience, because, as mentioned earlier, several studies (Erpelding et al., 2012; Emerson et al., 2014; Ruscheweyh et al., 2018) have demonstrated that MRI-derived local morphological characteristics are related to individual pain sensitivity. Second, MC is linked to DTI-derived structural connectivity and fMRI-derived functional connectivity, both of which are correlated with pain sensitivity, as mentioned earlier in this article (Wiech et al., 2014; Tu et al., 2019; Yuan et al., 2019; Spisak et al., 2020). The morphological covariance between brain regions reflects synchronized development (Alexander-Bloch et al., 2013a), given that the development trajectory of the structural covariance is correlated with the rate of change in cortical thickness and functional connectivity (Raznahan et al., 2011; Alexander-Bloch et al., 2013b). Moreover, Reid et al. (2017) have demonstrated that the extent of accordance between functional connectivity and MC varied remarkably across seed regions. Considering the close relationship between pain sensitivity and DTI-derived tractography or fMRI-derived functional connectivity, it is reasonable to hypothesize that MC is a possible neural determinant of pain sensitivity and MC can be used to establish a prediction model for individual pain sensitivity.

In the present study, we explored the relationship between MC and individual pain sensitivity based on MRI and behavioral data (pain thresholds) of 221 healthy participants. For each participant, two types of pain thresholds (laser and cold) were acquired as the measurements of pain sensitivity. We constructed the whole-brain MC networks for each participant and further used multivariate regression and feature selection methods to construct an MC-based prediction model and to find the most predictive MC features of pain sensitivity. Furthermore, to examine whether regional structural features (i.e., GMV) and MC could provide shared and complementary predictive information, we also built prediction models of pain sensitivity based on GMV features as well as based on both GMV and MC features. The performances of models based on MC, based on GMV, and based on both GMV and MC were compared to determine the best type of structural MRI features for the prediction of pain sensitivity.

## Materials and Methods

### Participants

We recruited a total of 221 healthy participants (135 females; age: 20.84 ± 2.81 years) through college and community advertisements and paid for their participation. All the participants were right-handed. Before the experiments, participants were carefully screened to ensure that they had no history of chronic pain, neurological diseases, cerebrovascular diseases, coronary heart disease, and mental disorders, and they had no contraindications to MRI examination. The study was proved by the local ethics committee, and all participants gave their written informed consent before participating in the study.

### Measurements of Pain Thresholds

Pain sensitivity of all the participants was measured as two types of pain thresholds (laser and cold) in two behavioral experiments before the MRI scan.

#### Laser Pain Threshold

The laser pain threshold was measured manually using quantitative sensory testing. A series of infrared neodymium yttrium aluminum perovskite (Nd: YAP) laser stimuli were delivered to the back area between the thumb and index finger of a participant’s left hand. The measurement was started from an energy level at 1 J with a 0.25 J increase at each stimulus. After each stimulus, a participant was asked to report the pain rating from 0 (no pain) to 10 (the worst pain). When a rating of 4 was reported, the corresponding energy level was recorded as the laser pain threshold. For each participant, the laser pain threshold was averaged from two independent measurements conducted in 1 h.

#### Cold Pain Threshold

The cold pain threshold was obtained from the cold pressor test. Each participant was instructed to complete the cold pressor test with his/her left hand. A participant immersed his/her left palm and upper arm in room temperature water (22 ± 0.5°C) for 30 s to eliminate the difference in hand temperature. Then, the participant was asked to quickly place the same hand into the cold water (2 ± 0.1°C). We recorded this moment as time point 1. Another moment when the participant started to feel pain was recorded as time point 2. The cold pain threshold was measured as the duration between the time the participant placed the hand on the cold pressor (time point 1) and the time when the participant first felt pain (time point 2).

#### Pain Sensitivity Score

We also summarized these two types of pain thresholds as one single composite measure of individual pain sensitivity. Laser pain threshold and cold pain threshold were normalized, and then their arithmetic mean was computed for each participant as his/her pain sensitivity score. A higher pain sensitivity score indicated lower pain sensitivity.

### MRI Acquisition

Structural MRI data were acquired using a GE 3.0 T scanner. High-resolution structural T1-weighted images were collected using a three-dimensional magnetization-prepared rapid gradient echo (3D-MPRAGE) sequence with following imaging parameters: 176 sagittal slices, time of echo (TE) = 2.992 ms, time of repetition (TR) = 6.896 ms, inversion time T1 = 450 ms, 1 mm slice thickness with no gap, acquisition matrix = 256 × 256, 1 × 1 mm in-plane resolution, acquisition time = 4.36 min.

### Data Analysis

#### Structural MRI Preprocessing

Structural MRI preprocessing was performed with SPM12 (Statistical Parametric Mapping; Wellcome Department of Imaging Neuroscience, University College London, United Kingdom)^1^ running under Matlab R2014a (Mathworks, Sherborn, MA). The structural images were segmented into GM, white matter, and cerebrospinal fluid by applying a registration to the MNI stereotactic space and a subsequent non-linear deformation. The non-linear deformation parameters were calculated via the inbuilt high dimensional Diffeomorphic Anatomical Registration Through Exponentiated Lie Algebra (DARTEL) algorithm (Ashburner, 2007). Then, the warping functions generated by DARTEL were used to spatially normalize the GM segments and modulate them by the Jacobian determinant. Finally, the segmented GM images were smoothed with an 8 mm full width at half maximum Gaussian kernel. The smoothed GM images of all participants were used for further analyses.

#### Brain Parcellation

The whole-brain parcellation was achieved by using the Automated Anatomical Labeling (AAL) atlas (Tzourio-Mazoyer et al., 2002). Cerebellar regions were excluded for incomplete coverage of the cerebellum of several participants. A total of 90 regions of interest (ROIs) were defined by the AAL atlas and used in subsequent analyses. Furthermore, when we used the Connectivity Visualization Tool^2^ to visualize the MC, the 90 ROIs were clustered into 16 lobes (prefrontal, motorstrip, insula, parietal, temporal, occipital, limbic, and subcortical in both left and right hemispheres) according to their coordinates. The details of 90 ROIs and 16 lobes were provided in Supplementary Table 1.

#### Feature Extraction and MC Estimation

In this study, we extracted two types of features, GMV and MC, from pre-processed GM images for the prediction of pain thresholds. GMV of each ROI was extracted as the local morphological feature. MC measures the inter-regional relations of local brain morphology at the individual level, and its calculation can be summarized in the following steps. First, the GM intensity at each voxel within one ROI was quantified in smoothed GM images. Second, the regional probability density function for each ROI was estimated using the kernel density estimation implemented in the Scipy package^3^. The kernel width was adaptively estimated from the data using Scott’s rule (Scott, 2010). Finally, the MC for each pair of ROIs was defined as the similarity between the two probability density functions of this pair of ROIs. In this work, the similarity of two brain regions was quantified by the Kullback–Leibler divergence. The Kullback–Leibler divergence was calculated as:

(1) $$KL\left(p,q\right)=\int_{X}\left(p\left(x\right)\log\left(\frac{p\left(x\right)}{q\left(x\right)}\right)+q\left(x\right)\log\left(\frac{q\left(x\right)}{p\left(x\right)}\right)\right),$$

where *p*(*x*) and *q*(*x*) were the probability density functions of two ROIs *p* and *q*. The MC matrix for each individual was a 90 × 90 symmetric matrix. Only the lower triangular matrix, which has 4005 MC features, was taken for subsequent analysis. Furthermore, we conducted the correlation analysis between the MC features and GMV features to find the relationship between these features.

### Prediction of Pain Thresholds

After local morphological characteristics (i.e., GMV) and structural connectivity (i.e., MC) were extracted from structural MRI, we used feature selection and machine learning techniques to identify features that are most predictive of pain thresholds and to establish models for predicting pain thresholds from GMV and MC features. The whole procedure of pain threshold prediction analysis includes the following steps.

#### Model Development

We used a popular and effective multivariate regression method, partial least squares regression (PLSR), to model the relationship between high-dimensional structural MRI features (GMV, MC, or their concatenation) and one of three types of pain thresholds (laser, cold, or their summary, pain sensitivity score). The PLSR model is formulated as:

(2) y=Xα+E

where **y** is an *N*×1 vector containing one type of pain threshold of *N* participants, **X** is an *N*×*K* matrix consisting of *K* structural MRI features (GMV, MC, or their concatenation) of *N* participants, α is the *K*×1 PLSR coefficient vector (of which each entry denotes the contribution of the corresponding feature to the prediction result), and **E** is the error term.

In the PLSR model of Eq. (2), features could be the mean GMV of all voxels in each ROI (*K* = 90), the whole-brain MCs (*K* = 4,005), or the concatenation of these two types of features (GMV+MC, *K* = 4,095). The labels of the prediction model could be the laser pain thresholds, the cold pain thresholds, or the composite pain sensitivity scores. The SIMPLS algorithm (De Jong, 1993) was used to compute the PLSR model coefficients. The number of latent components in the PLSR analysis was estimated using the coefficient of determination, which calculates the percentage of the variance of the values fitted by the latent components and the total variance of the dependent variables.

#### Feature Selection

There are two types of candidate features, GMV and MC, for the prediction of pain thresholds. To compare the model performance under different types of features, we first separately used GMV and MC as features in the prediction analysis and then used a combination of GMV and MC features for prediction. Further, it is necessary to identify a subset of most predictive and discriminative features from a large number of features (especially for MC features), because feature selection can improve the model accuracy and can increase the model interpretability. Considering that each coefficient in the PLSR coefficient vector represents the predictive capability of the corresponding feature, we ranked all features in a descending order according to their absolute values and then selected a certain number of features with large magnitudes as the feature subset for prediction. We compared the model accuracy with different numbers of features and finally selected the number of features with the highest accuracy to build the prediction model. To improve the efficiency of the feature selection operation for high-dimensional MC features (*K* = 4,005), we first examined the model accuracy under different numbers of features from 1 to 4,005 with a searching interval of 100. After we used the coarse-scale search to find the “optimal” number of features, which had the highest accuracy under a searching interval of 100 and was denoted as *m*_*100*_, we started a new round of search in a narrower range from (*m*_100_−100) to (*m*_100_ + 100) and with a smaller searching interval of 10. Such a feature selection procedure was repeated with a decreasing searching interval (100, 10, 1) until the exact number of features was determined. For GMV, the feature selection procedure was similar to that of MC, but only two searching intervals, 10 and 1, were used. Note that, because we used leave-one-individual-out cross-validation (see below for details) to train and test the pain prediction model, the ranked PLSR coefficients and the selected features are different for each participant. To select features at the group level (i.e., to select the same subset of features for all participants), we averaged PLSR coefficient vectors of all participants, ranked the averaged coefficients, and then found the subset of features with the highest prediction accuracy at the group level. The BrainNet viewer^4^ (Xia et al., 2013) and Brain Connectivity Toolbox^5^ (Shen et al., 2017) were used to visualize the selected GMV features and MC features.

#### Cross-Validation

We trained and tested the PLSR models based on the leave-one-individual-out cross-validation. At each run, we randomly used one participant’s data for testing and the remaining participants’ data for training. Because we had a total of 221 participants, the procedure was repeated 221 times to make sure that each participant’s data were used as test samples for once.

#### Performance Evaluation

The accuracy of these prediction models was measured by two metrics: mean absolute error (MAE) and mean relative absolute error (MRAE). MAE and MRAE are, respectively, calculated as:

(3) $$MAE=\frac{1}{N}\sum_{i=1}^{N}\left|\hat{y_{i}}-y_{i}\right|,$$

(4) $$MRAE=\frac{1}{N}\sum_{i=1}^{N}\left|\frac{\hat{y_{i}}-y_{i}}{y_{i}}\right|,$$

where *y_i* is the measured pain threshold of the *i*th participant, $\hat{y_{i}}$ is the pain threshold estimated from the PLSR model, and *N* is the total number of individuals. MAE is a measure of the overall distance between predicted and true values, while MRAE expresses how large the absolute error is as compared with the true values. We used both MAE and MRAE for performance comparison because they had their own limitations: MAE was dependent on the magnitude of pain thresholds while MRAE could be influenced by small values close to zero (which was true for the pain sensitivity score). Further, we calculated Pearson’s correlation coefficients between the predicted thresholds and the true values across all participants, because Pearson’s correlation coefficients were not influenced by the range of pain thresholds and data normalization. The prediction performance of models with different types and numbers of features was compared using a two-sided Wilcoxon rank-sum test.

## Results

### Measurements of Pain Thresholds

For all participants, the laser pain thresholds were 2.58 ± 0.53 (mean ± *SD*), the cold pain thresholds were 9.59 ± 0.38, and the pain sensitivity scores were 0.41 ± 0.14. We calculated Pearson’s correlation coefficients between age and different types of pain sensitivity thresholds, but found no significant relationship between age and any pain threshold (*P* > 0.05 for all correlations). Similarly, a two-sample *t*-test revealed that gender had no significant effect on pain thresholds (*P* > 0.05 for all tests). Further, we calculated Pearson’s correlation coefficients of three types of thresholds and found that each pair was significantly correlated (laser pain threshold vs. cold pain threshold: *R* = 0.22, *P* = 0.0011; laser pain threshold vs. pain sensitivity score: *R* = 0.76, *P* < 10^–10^; cold pain threshold vs. pain sensitivity score: *R* = 0.71, *P* < 10^–10^).

### Prediction of Pain Thresholds

**FIGURE 1:**
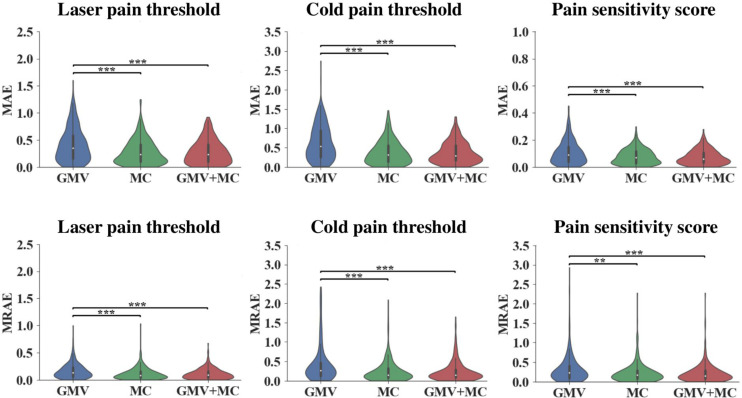
Prediction errors of using different feature sets (GMV, MC, and GMV + MC) to predict three types of pain thresholds (laser pain threshold, cold pain threshold, and pain sensitivity score). **Indicates *P* < 0.01, *** indicates *P* < 0.001, two-sided Wilcoxon rank-sum test.

**FIGURE 2:**
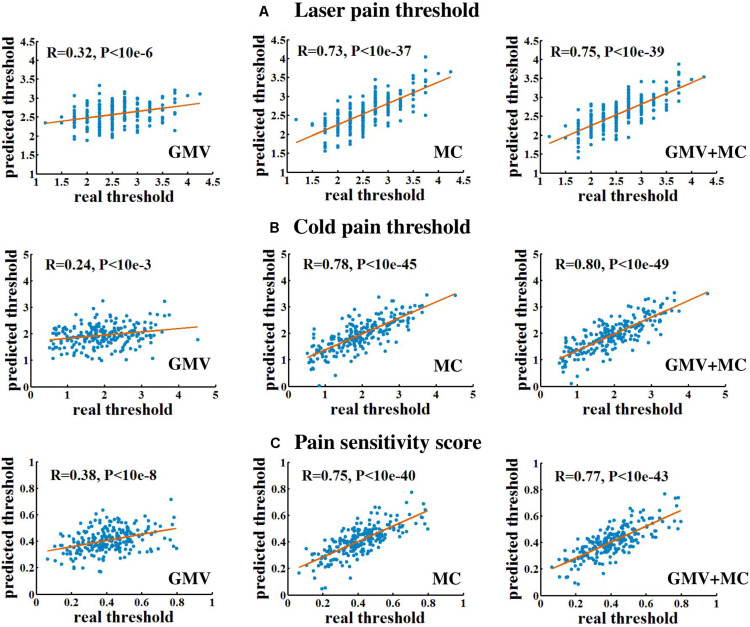
The linear correlation between predicted and real pain thresholds. **(A)** Prediction of laser pain threshold. **(B)** Prediction of cold pain threshold. **(C)** Prediction of pain sensitivity score. Each blue dot denotes one participant. Red lines are linear fitting lines.

Figure 1 and Table 1 show the performance of pain threshold prediction based on three different sets of features: GMV, MC, and GMV+MC (the concentration of the GMV feature vector and the MC feature vector). We have the following three major observations from Figure 1 and Table 1. First, prediction errors based on MC were significantly lower than those based on GMV, no matter which type of pain threshold was predicted. Second, prediction errors based on GMV+MC were also significantly lower than those based on GMV for all pain thresholds. Third, although prediction errors based on GMV + MC were slightly lower than those based on MC only for all pain thresholds, there was no significant difference between prediction errors of MC and GMV + MC. Fourth, we can see that laser pain threshold showed higher MAE but lower MRAE than pain sensitivity score, which should be due to the different ranges of laser thresholds and pain sensitivity scores. The range of pain sensitivity score was 0–1, and many scores were close to 0. According to the calculation of MRAE, a very small pain sensitivity score (close to 0) will lead to a very large MRAE. On the other hand, the range of laser threshold is 1.75–4.25, which is not close to zero. As a result, MRAE of laser pain threshold did not have very large values, and it was smaller than MRAE of pain sensitivity score. Figure 2 shows the linear correlations between predicted and real pain thresholds of all the participants in different prediction models, and the correlation values were provided in Table 1. Correlation results of using all types of features for all types of pain threshold measure are significant. The prediction performance in terms of correlation coefficients based on MC or GMV + MC was significantly better than that based on GMV, no matter which type of pain threshold was predicted. However, there was no significant difference between the correlation coefficients based on MC and GMV + MC (*P* = 0.322 for laser pain threshold, *P* = 0.289 for cold pain threshold, and *P* = 0.310 for pain sensitivity score).

**TABLE 1:** Prediction performance in predicting three types of pain thresholds using three feature sets.

	Feature set	MAE (mean ± *SD*)	MRAE (mean ± *SD*)	Correlation coefficient
Laser pain threshold	MC	0.28 ± 0.23	0.11 ± 0.11	0.73
	GMV	0.40 ± 0.32	0.16 ± 0.15	0.32
	MC+GMV	0.28 ± 0.22	0.11 ± 0.10	0.75
Cold pain threshold	MC	0.38 ± 0.30	0.25 ± 0.30	0.78
	GMV	0.62 ± 0.46	0.42 ± 0.48	0.24
	MC+GMV	0.37 ± 0.28	0.24 ± 0.26	0.80
Pain sensitivity score	MC	0.08 ± 0.06	0.23 ± 0.25	0.75
	GMV	0.11 ± 0.09	0.33 ± 0.41	0.38
	MC+GMV	0.07 ± 0.05	0.22 ± 0.25	0.77

### Predictive GMV and MC Features

**FIGURE 3:**
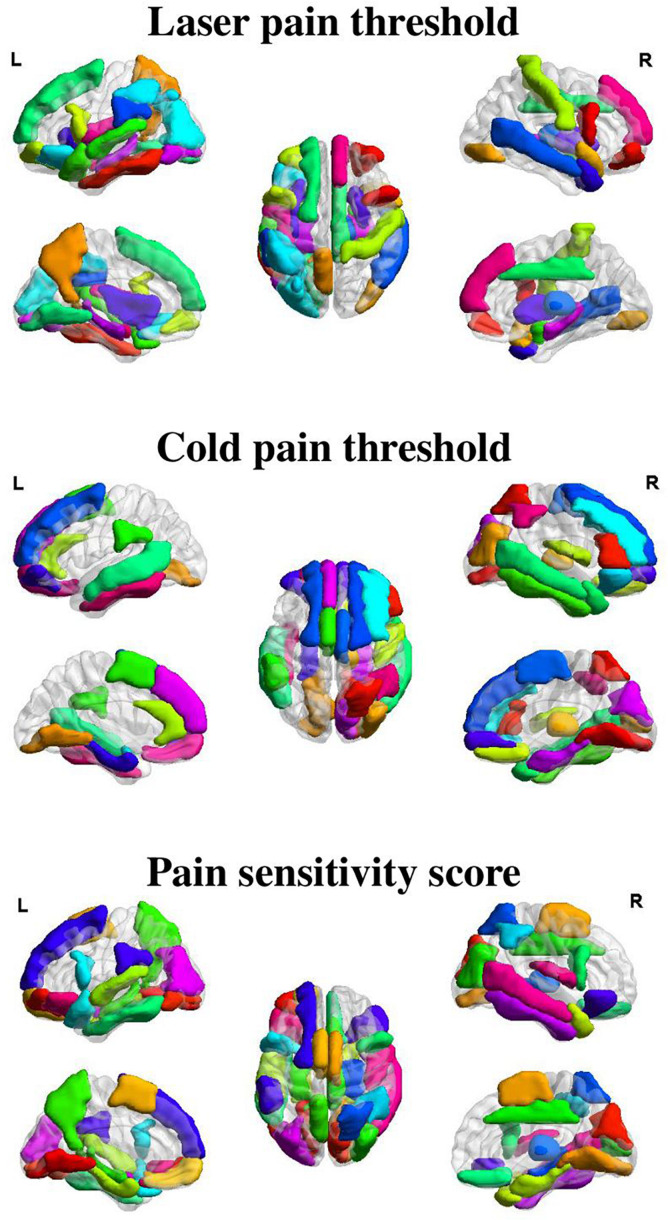
Selected GMV features for the prediction of different measures of pain sensitivity. There were 38, 32, and 41 GMV feature regions selected for the prediction of laser pain thresholds, cold pain thresholds, and pain sensitivity scores, respectively.

**FIGURE 4:**
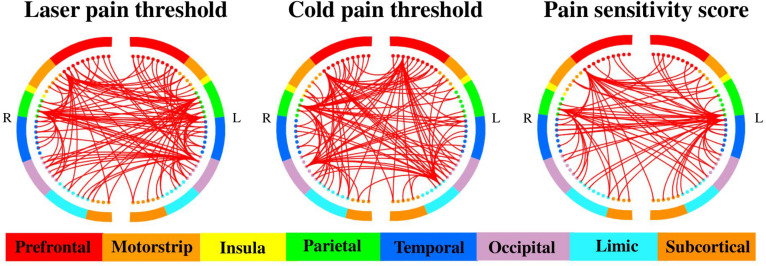
Selected MC features for the prediction of different measures of pain sensitivity. There were 460, 508, and 485 MC features selected for the prediction of laser pain thresholds, cold pain thresholds, and pain sensitivity scores, respectively. The two half circles represent two brain hemispheres, and the red lines represent the MC between two corresponding regions.

Figures 3, 4 show the optimal GMV and MC feature subsets, respectively, selected for pain threshold prediction. According to the coefficient ranking in PLSR models, we selected different numbers of features to make predictions and the selected optimal feature set could achieve the best prediction performance. Supplementary Figure 1 shows the prediction error of the model built with different numbers of features for the prediction of laser pain threshold, cold pain threshold, and pain sensitivity score.

As shown in Figure 3, GMV features of 38 ROIs were selected for prediction of laser pain thresholds, and they were mainly in the PFC, precuneus, temporal lobe, median cingulate cortex, thalamus, and hippocampus; GMV features of 32 ROIs were selected for prediction of cold pain thresholds, and they were mainly in the PFC, temporal lobe, lingual gyrus, supplementary motor area, and parahippocampus gyrus; GMV features of 41 ROIs were selected for prediction of pain sensitivity scores, and they were mainly in the PFC, precuneus, thalamus, temporal lobe, and lingual gyrus.

On the other hand, as shown in Figure 4, there were 460, 508, and 485 MC features selected for prediction of laser pain thresholds, cold pain thresholds, and pain sensitivity scores, respectively. According to the number of selected MC features in each pair of lobes, we listed those pairs of lobes having the top 5 largest number of selected MC features in Table 2. Supplementary Tables 2–4 provide complete results about the selected MC features in each pair of lobes.

**TABLE 2:** List of pairs of lobes having most selected MC features for the prediction of different measures of pain sensitivity.

Laser pain threshold	Cold pain threshold	Pain sensitivity score
Prefrontal_L–Occipital_R	Prefrontal_L–Prefrontal_R	Prefrontal_L–Prefrontal_R
Prefrontal_L–Occipital_L	Prefrontal_R–Occipital_L	Temporal_L–Temporal_R
Occipital_L–Occipital_R	Prefrontal_R–Temporal_L	Prefrontal_L–Parietal_L
Prefrontal_L–Prefrontal_R	Prefrontal_L–Occipital_L	Prefrontal_L–Temporal_R
Prefrontal_L–Parietal_L	Prefrontal_R–Occipital_R	Prefrontal_L–Occipital_R
Prefrontal_R–Parietal_L	Prefrontal_R–Subcortical_L	Prefrontal_R–Temporal_L
Prefrontal_R–Occipital_R	Temporal_L–Temporal_R Occipital_L–Occipital_R	

*Those pair of lobes having the top five largest number of selected MC features are listed in this table. Because some pairs of lobes have the same number of selected features or the same selection ration, more than five pairs of lobes are listed for the prediction of each measure of pain sensitivity.*

Furthermore, Table 3 shows the common and different pairs of lobes having most selected MC features for the prediction of laser and cold pain thresholds. Note that MC features selected for the prediction of pain sensitivity score were not considered in Table 3, because pain sensitivity score is a composite measure of both laser and cold pain thresholds. It can be seen from Table 3 that MC features within prefrontal lobes, within occipital lobes, and between prefrontal and occipital lobes are predictive of both laser and cold pain thresholds, while laser and cold pain thresholds also have some specific predictive MC patterns. For example, MC related to the parietal lobes may be more predictive of laser pain threshold, while MC related to the temporal lobes may be more predictive of cold pain threshold. Importantly, we can see from Table 3 that the prefrontal lobes (i.e., PFC) are involved in almost all listed pairs of lobes with most predictive MC features, suggesting the key role of PFC in the study of pain sensitivity.

**TABLE 3:** List of common and different pairs of lobes having most selected MC features for the prediction of different measures of pain sensitivity.

Both laser and cold pain thresholds	Laser pain thresholds only	Cold pain thresholds only
Prefrontal_L–Occipital_L	Prefrontal_L–Occipital_R	Prefrontal_R–Occipital_L
Occipital_L–Occipital_R	Prefrontal_L–Parietal_L	Prefrontal_R–Temporal_L
Prefrontal_L–Prefrontal_R	Prefrontal_R–Parietal_L	Prefrontal_R–Subcortical_L
Prefrontal_R–Occipital_R		Temporal_L–Temporal_R

## Discussion

In this study, we investigated the predictive power of the brain’s inter-regional structural connections, as measured by MC, in the prediction of individual pain sensitivity, as measured by three types of pain thresholds, laser pain threshold, cold pain threshold, and their summarized score. Our results revealed that MC is capable of accurately predicting an individuals’ pain thresholds and, importantly, its predictive capability is significantly higher than the brain’s regional morphological features (i.e., GMV). Therefore, MC can provide a new insight into the neural basis of pain sensitivity, and it holds the potential in predicting an individual’s pain sensitivity in clinical practice.

### MC as New Neural Correlates of Pain Sensitivity

Previous studies have shown that MRI-based regional morphological features can be used to predict pain sensitivity (Erpelding et al., 2012; Emerson et al., 2014; Ruscheweyh et al., 2018). However, there is still no study exploring the individual-level relationship between pain sensitivity and MC, which contains the brain’s higher-order structural cortical information. Because pain sensitivity is related to a wide network of brain regions, using brain connectivity measures to predict pain sensitivity is promising. Considering resting-state functional connectivity has been used in the prediction of individual pain thresholds (Tu et al., 2019; Spisak et al., 2020) and there is a close relationship between functional connectivity and MC (Reid et al., 2017), we hypothesized that MC could be a new type of MRI-based neural predictors of individual pain sensitivity.

Although MC has not been used to correlate pain sensitivity in literature, it is widely used as a measure of individual-level structural connectivity to investigate perception, cognition and neurological disorders (Li and Kong, 2017; Wang et al., 2018; Wang et al., 2020). However, the physiological meaning underlying inter-regional MC is complex and has not been completely understood. There are two possible explanations for MC. One stems from the axon tension theory (Van Essen, 1997), which proposes that anatomically connected brain areas are pulled by a mechanical force, resulting in similar morphological properties. Another explanation is that regions with similar morphological distributions might reflect coordination between areas in development (Lerch et al., 2006; Alexander-Bloch et al., 2013b) and learning (Draganski et al., 2004; Mechelli et al., 2004).

The performance of the proposed prediction models based on MC features is satisfactory and encouraging among similar MRI-based pain sensitivity prediction models in literature, despite the features and the type of pain threshold used in these prediction models are different. Tu et al. (2019) predicted the heat pain thresholds of 24 individuals from resting-state fMRI connectome, and they achieved a correlation coefficient of 0.60 between predicted and real pain thresholds. Spisak et al. (2020) used resting-state functional connectivity to achieve a correlation coefficient of 0.63 between predicted and real values of pain sensitivity scores, which were composed of heat, cold, and mechanical pain thresholds, of a total of 116 individuals. Another study (Schulz et al., 2012) applied multivariate pattern analysis on time–frequency transformed single-trial EEG responses to predict individuals’ pain sensitivity, which was measured by a simplified dichotomous model, and it achieved an accuracy of 83% in classifying individuals with high pain sensitivity and with low pain sensitivity. Although these predictive models cannot be directly compared because they used different features and measures of pain sensitivity, we can still see that our proposed MC-based model has excellent and promising results (for example, correlation coefficients between actual and predicted pain thresholds > 0.73) in continuous prediction of different measures of pain sensitivity.

### MC Features Predictive of Pain Sensitivity

We first briefly discussed the selected GMV features. According to the ranking of prediction coefficients, we found that GMV in the PFC have higher predictive power in the prediction of all three measures of pain sensitivity. As identified in the previous studies, PFC is the key region that underlies executive functions such as planning, problem solving, and social control (Goldman-Rakic, 1996). A study (Lorenz et al., 2003) suggested that the PFC may exert active control on pain perception by modulating cortico-subcortical and cortico-cortical pathways. Also, Coghill et al. (2003) found that the pain-induced BOLD responses of the PFC are positively related to individual pain sensitivity, suggesting that PFC is both structurally and functionally correlated with pain sensitivity. In addition, there is evidence that patients with chronic low back pain have regional GM alterations in the PFC (Apkarian et al., 2004; Schmidt-Wilcke et al., 2006; Seminowicz et al., 2011; Ivo et al., 2013; Ung et al., 2014).

As for pain-predictive MC features, we finally selected around 500 MC features for the prediction of each measure of pain sensitivity. By ranking pairs of lobes according to the number of selected MC features they had, we found that some PFC-related MC features, such as those of prefrontal–occipital and prefrontal_L–prefrontal_R are most predictive in the prediction of both laser and cold pain thresholds. These results further confirmed the crucial role of PFC in the determination of pain sensitivity. Not only the local structural features, but also the MC features of PFC are able to accurately predict the level of pain sensitivity. Apart from these shared predictive connections, we observed different patterns of predictive MC for different pain thresholds. For example, prefrontal–parietal MCs are more effective in the prediction of laser pain threshold than in the prediction of cold pain threshold. On the other hand, MCs between temporal and prefrontal are more useful in the prediction of cold pain threshold. As discussed in previous studies (Dubin and Patapoutian, 2010; Tu et al., 2019), the heat pain and cold pain are mediated by partially different sensory pathways; this may cause the difference in selected MC features in prediction of different pain thresholds.

By comparing the prediction performance of models based on MC and GMV, we found that MC features are more predictive than GMV features. However, the predictive performance of combining these two kinds of features is not significantly higher than that of only using MC. The reason may be that some predictive MC features and GMV features are highly correlated. Supplementary Figure 2 shows the cross-participant correlation between MC features and GMV features. We can see that, for example, frontal–subcortical MCs, which are found to be predictive of pain sensitivity in the present study, are significantly related to GMV features of almost all regions. Therefore, MC and GMV features may provide shared but not complementary predictive information. Also, when we used the combination of these two types of features for prediction, the feature subset we finally selected were mostly MC features. So, the combination of GMV and MC features provided no more predictive information than only MC features did. These results suggested that MC could provide exclusive useful information about the mechanism of pain sensitivity, and such information cannot be extracted from local morphological measures.

### Limitations and Future Work

Some limitations of the present study are mentioned here. First, the cerebellum was not included in the whole-brain morphological analysis because several participants had incomplete coverage of the cerebellum. Previous studies have suggested that the cerebellum has a role in pain and nociceptive processing (Moulton et al., 2010; Tu et al., 2019), so MC between cerebellum and other regions may also be predictive of pain thresholds. Second, the AAL atlas, which was widely used in structural MRI studies, was used in our study to construct MC. However, the prediction performance could be affected by the selection of atlases in the quantification of structural brain connectivity. It is not easy to ascertain the most suitable atlas, so it would be useful to compare the prediction results by using different atlas-based structural connectivity. Further, previous studies (Min et al., 2014; Zeng et al., 2018) have demonstrated that methods using multiple atlases could outperform those using a single atlas. For example, Min et al. proposed a data-driven atlas selection scheme to obtain the most distinctive and representative atlases, and the proposed multi-atlas-based method achieved a significantly better performance (91.64%) than the methods that only used one atlas (87.05%) in Alzheimer’s disease diagnosis. Thus, it would be possible to use multiple atlases and associated multi-atlas-based methods to further improve the prediction performance. Third, the prediction analysis was based on data from only one site, so the developed MC-based prediction models may have problems of replicability, generalizability, and overfitting. It is desired to use data from multiple and independent sites for model validation. Fourth, pain sensitivity and brain structural networks change with age, but this study only acquired data from young people aged from 17 to 28. Thus, it would be useful to study the stability of pain characteristics in a cohort with more diversified demographical characteristics, such as age. Fifth, the regression model used in this study to predict pain thresholds is based on the simple PLSR. We also used another popular regression model, support vector regression (SVR), to predict pain thresholds, and the SVR-based results (prediction accuracy and MC features selected) were similar to those based on PLSR (see Supplementary Table 5 and Supplementary Figure 3 for details). It may also be possible to use deep neural networks to construct the prediction model, but deep neural networks have difficulty in producing interpretable features and their performance are limited by the number of data samples. In the future, it is possible and desired to develop and apply deep learning models with good interpretability on “big” pain neuroimaging data to predict pain thresholds. Finally, because the exact physiological meaning of individual-level MC is still unclear, it is necessary to combine different MRI modalities, such as fMRI and DTI, and medical data, such as gene expression data, to explore the mechanism of pain sensitivity from different and complementary perspectives.

## Conclusion

In conclusion, this study used T1-weighted inter-regional MC as a new type of feature to predict individual pain sensitivity as measured by laser and cold pain thresholds and found that a set of MC features could provide more predictive power than local morphological characteristics. Examining the relationship between MC and pain sensitivity is important for a better understanding of pain’s neural mechanisms, and our finding may facilitate the development of individualized pain treatment and management.

## Data Availability Statement

The datasets presented in this article are not readily available because this dataset is not publicly accessible. We will consider sharing later. Requests to access the datasets should be directed to ZZ, zgzhang@szu.edu.cn.

## Ethics Statement

The studies involving human participants were reviewed and approved by the Ethics Committee of Institute of Psychology, Chinese Academy Sciences, the Ethics Committee of Liaoning Normal University, and the Ethics Committee of Shenzhen University.

## Author Contributions

RZ, LL, and ZZ contributed to the construction of the study hypothesis. GH and ZL collected the data. RZ analyzed the data. RZ, LL, LZ, and ZZ discussed the results and wrote the manuscript. All authors contributed to the article and approved the submitted version.

## Conflict of Interest

The authors declare that the research was conducted in the absence of any commercial or financial relationships that could be construed as a potential conflict of interest.

## Footnotes

1 http://www.fil.ion.ucl.ac.uk/spm

2 https://bioimagesuiteweb.github.io/webapp/connviewer.html

3 http://www.scipy.org/

4 http://www.nitrc.org/projects/bnv/

5 http://www.brain-connectivity-toolbox.net/
