# Supplementary material for: Predicting Individual Pain Thresholds From Morphological Connectivity Using Structural MRI: A Multivariate Analysis Study
Source: Front Neurosci. 2021 Feb 10;15:615944. doi: 10.3389/fnins.2021.615944 (PMC7902866; doi:10.3389/fnins.2021.615944)
Supplement: Supplementary file 1 [file Data_Sheet_1.PDF]

Supplemental Materials

1. List of ROIs and Lobes

In this study, 90 ROIs were defined by the AAL atlas. Furthermore, when using the Connectivity Visualization Tool (https://bioimagesuiteweb.github.io/webapp/connviewer.html) to visualize the MC, the 90 ROIs were clustered into 16 lobes (prefrontal, motorstrip, insula, parietal, temporal, occipital, limbic and subcortical in both left and right hemispheres) according to their coordinates. The details of 90 ROIs and 16 lobes were provided in Supplementary Table 1.

Supplementary Table 1. List of 90 ROIs and 16 lobes

Left Hemisphere		Right Hemisphere	
**Lobe**	**ROI**	**Lobe**	**ROI**
prefrontal	Frontal_Sup_L<br>Frontal_Sup_Orb_L<br>Frontal_Mid_L<br>Frontal_Mid_Orb_L<br>Frontal_Inf_Oper_L<br>Frontal_Inf_Tri_L<br>Frontal_Inf_Orb_L<br>Frontal_Sup_Medial_L<br>Frontal_Mid_Orb_L<br>Rectus_L	prefrontal	Frontal_Sup_R<br>Frontal_Sup_Orb_R<br>Frontal_Mid_R<br>Frontal_Mid_Orb_R<br>Frontal_Inf_Oper_R<br>Frontal_Inf_Tri_R<br>Frontal_Inf_Orb_R<br>Frontal_Sup_Medial_R<br>Frontal_Mid_Orb_R<br>Rectus_R
motorstrip	Precentral_L<br>Rolandic_Oper_L<br>Supp_Motor_Area_L<br>Paracentral_Lobule_L	motorstrip	Precentral_R<br>Rolandic_Oper_R<br>Supp_Motor_Area_R<br>Postcentral_R<br>Paracentral_Lobule_R
insula	Insula_L	insula	Insula_R
parietal	Postcentral_L<br>Parietal_Sup_L<br>Parietal_Inf_L<br>SupraMarginal_L<br>Angular_L<br>Precuneus_L	parietal	Parietal_Sup_R<br>Parietal_Inf_R<br>SupraMarginal_R<br>Angular_R
temporal	Fusiform_L<br>Heschl_L<br>Temporal_Sup_L<br>Temporal_Pole_Sup_L	temporal	Fusiform_R<br>Heschl_R<br>Temporal_Sup_R<br>Temporal_Pole_Sup_R

	Temporal_Mid_L<br>Temporal_Pole_Mid_L<br>Temporal_Inf_L		Temporal_Mid_R<br>Temporal_Pole_Mid_R<br>Temporal_Inf_R
occipital	Calcarine_L<br>Cuneus_L<br>Lingual_L<br>Occipital_Sup_L<br>Occipital_Mid_L<br>Occipital_Inf_L	occipital	Calcarine_R<br>Cuneus_R<br>Lingual_R<br>Occipital_Sup_R<br>Occipital_Mid_R<br>Occipital_Inf_R
limbic	Cingulum_Ant_L<br>Cingulum_Mid_L<br>Cingulum_Post_L<br>Hippocampus_L<br>ParaHippocampal_L<br>Amygdala_L	limbic	Cingulum_Ant_R<br>Cingulum_Mid_R<br>Cingulum_Post_R<br>Hippocampus_R<br>ParaHippocampal_R<br>Amygdala_R<br>Precuneus_R
subcortical	Olfactory_L<br>Caudate_L<br>Putamen_L<br>Pallidum_L<br>Thalamus_L	subcortical	Olfactory_R<br>Caudate_R<br>Putamen_R<br>Pallidum_R<br>Thalamus_R

2. MC features predictive of pain sensitivity

In this study, we selected a small subset of optimal MC features for the prediction of three measures of pain sensitivity (laser pain threshold, cold pain threshold, and pain sensitivity score). Supplementary Figure 1 shows the prediction error of the model built with different number of features for the prediction of laser pain threshold, cold pain threshold, and pain sensitivity score. Supplementary Tables 2, 3 and 4 show the number of selected MC features in each pair of lobes for the prediction of laser pain threshold, cold pain threshold, and pain sensitivity score, respectively.

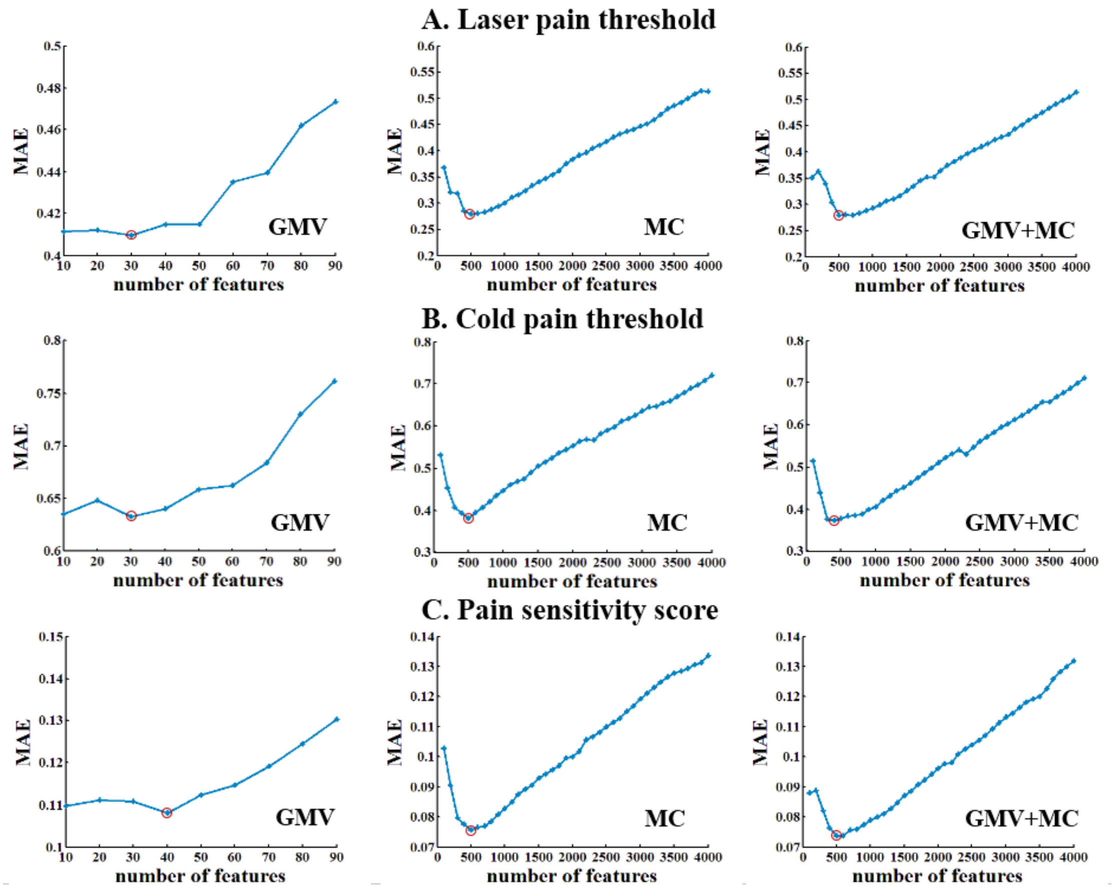

Supplementary Figure 1. The prediction error of the model built with different number of features. A. Prediction of laser pain threshold. **B.** Prediction of cold pain threshold. **C.** Prediction of pain sensitivity score.

Supplementary Table 2. The number of selected MC features for each pair of lobes in the prediction of laser pain threshold

Lobe	Prefrontal_L	Prefrontal_R	Motorstrip_L	Motorstrip_R	Insula_L	Insula_R	Parietal_L	Parietal_R	Temporal_L	Temporal_R	Occipital_L	Occipital_R	Limbic_L	Limbic_R	Subcortical_L	Subcortical_R
**Prefrontal_L**	6	**12**	3	5	2	2	**12**	7	10	9	**15**	**17**	1	2	1	3
**Prefrontal_R**	**12**	4	8	3	2	0	**12**	5	11	7	10	**12**	6	6	2	1
**Motorstrip_L**	3	8	0	5	1	0	3	2	1	2	7	3	0	1	0	1
**Motorstrip_R**	5	3	5	1	2	0	8	3	4	0	3	1	0	1	1	2
**Insula_L**	2	2	1	2	0	0	0	1	1	0	1	1	0	0	0	1
**Insula_R**	2	0	0	0	0	0	0	0	1	1	1	0	0	1	2	0
**Parietal_L**	**12**	**12**	3	8	0	0	5	7	4	8	5	6	3	2	2	2
**Parietal_R**	7	5	2	3	1	0	7	1	5	5	6	10	1	2	0	0
**Temporal_L**	10	11	1	4	1	1	4	5	4	9	9	7	4	4	5	2
**Temporal_R**	9	7	2	0	0	1	8	5	9	3	6	3	2	4	4	2
**Occipital_L**	**15**	10	7	3	1	1	5	6	9	6	4	**13**	5	5	4	2
**Occipital_R**	**17**	**12**	3	1	1	0	6	10	7	3	**13**	2	0	1	3	1
**Limbic_L**	1	6	0	0	0	0	3	1	4	2	5	0	0	5	2	2
**Limbic_R**	2	6	1	1	0	1	2	2	4	4	5	1	5	2	3	1
**Subcortical_L**	1	2	0	1	0	2	2	0	5	4	4	3	2	3	1	1
**Subcortical_R**	3	1	1	2	1	0	2	0	2	2	2	1	2	1	1	2

According to Supplementary Table 2, MC features that are predictive of laser pain thresholds were mainly in the following pairs of lobes (which have top 5 largest number of selected MC features): Prefrontal_L-Occipital_R, Prefrontal_L-Occipital_L, Occipital_L-Occipital_R, Prefrontal_L-Prefrontal_R, Prefrontal_L-Parietal_L, Prefrontal_R-Parietal_L, Prefrontal_R-Occipital_R.

Supplementary Table 3. The number of selected MC features for each pair of lobes in the prediction of cold pain threshold

Lobe	Prefrontal_L	Prefrontal_R	Motorstrip_L	Motorstrip_R	Insula_L	Insula_R	Parietal_L	Parietal_R	Temporal_L	Temporal_R	Occipital_L	Occipital_R	Limbic_L	Limbic_R	Subcortical_L	Subcortical_R
**Prefrontal_L**	5	**18**	5	7	0	1	8	9	8	8	**10**	9	3	8	7	3
**Prefrontal_R**	**18**	7	5	9	1	0	8	7	**10**	7	**12**	**10**	8	9	**10**	6
**Motorstrip_L**	5	5	1	4	0	0	3	3	2	1	2	4	2	1	0	0
**Motorstrip_R**	7	9	4	4	0	0	4	3	1	3	2	1	0	1	0	0
**Insula_L**	0	1	0	0	0	0	0	1	1	0	2	1	1	1	2	0
**Insula_R**	1	0	0	0	0	0	0	0	2	1	1	0	1	1	2	1
**Parietal_L**	8	8	3	4	0	0	3	4	8	4	7	9	1	3	1	1
**Parietal_R**	9	7	3	3	1	0	4	0	5	3	5	6	4	6	0	1
**Temporal_L**	8	**10**	2	1	1	2	8	5	1	**10**	9	9	8	5	4	1
**Temporal_R**	8	7	1	3	0	1	4	3	**10**	2	8	6	9	4	3	4
**Occipital_L**	**10**	**12**	2	2	2	1	7	5	9	8	3	**10**	8	5	3	2
**Occipital_R**	9	**10**	4	1	1	0	9	6	9	6	**10**	2	6	4	4	3
**Limbic_L**	3	8	2	0	1	1	1	4	8	9	8	6	0	3	6	4
**Limbic_R**	8	9	1	1	1	1	3	6	5	4	5	4	3	0	4	2
**Subcortical_L**	7	**10**	0	0	2	2	1	0	4	3	3	4	6	4	2	4
**Subcortical_R**	3	6	0	0	0	1	1	1	1	4	2	3	4	2	4	1

According to Supplementary Table 3, MC features that are predictive of cold pain thresholds were mainly in the following pairs of lobes (which have top 5 largest number of selected MC features): Prefrontal_L-Prefrontal_R, Prefrontal_R-Occipital_L, Prefrontal_R-Temporal_L, Prefrontal_L-Occipital_L, Prefrontal_R-Occipital_R, Prefrontal_R-Subcortical_L, Temporal_L-Temporal_R, Occipital_L-Occipital_R.

Supplementary Table 4. The number of selected MC features for each pair of lobes in the prediction of pain sensitivity score

Lobe	Prefrontal_L	Prefrontal_R	Motorstrip_L	Motorstrip_R	Insula_L	Insula_R	Parietal_L	Parietal_R	Temporal_L	Temporal_R	Occipital_L	Occipital_R	Limbic_L	Limbic_R	Subcortical_L	Subcortical_R
**Prefrontal_L**	5	**14**	5	9	2	1	**11**	6	10	**11**	9	**11**	1	0	4	3
**Prefrontal_R**	**14**	3	9	7	1	0	9	7	**11**	7	9	7	6	6	4	5
**Motorstrip_L**	5	9	1	5	0	0	3	1	1	1	4	2	0	1	0	0
**Motorstrip_R**	9	7	5	3	0	0	7	1	3	3	4	2	0	2	0	2
**Insula_L**	2	1	0	0	0	0	1	1	1	0	1	1	1	0	1	1
**Insula_R**	1	0	0	0	0	0	0	0	1	1	1	0	1	0	0	0
**Parietal_L**	**11**	9	3	7	1	0	2	8	7	5	9	7	2	5	0	1
**Parietal_R**	6	7	1	1	1	0	8	1	2	3	7	9	3	5	1	2
**Temporal_L**	10	**11**	1	3	1	1	7	2	2	**13**	8	6	7	3	7	1
**Temporal_R**	**11**	7	1	3	0	1	5	3	**13**	3	9	4	7	7	6	4
**Occipital_L**	9	9	4	4	1	1	9	7	8	9	4	10	6	4	3	2
**Occipital_R**	**11**	7	2	2	1	0	7	9	6	4	10	4	6	2	3	2
**Limbic_L**	1	6	0	0	1	1	2	3	7	7	6	6	0	5	3	2
**Limbic_R**	0	6	1	2	0	0	5	5	3	7	4	2	5	1	6	1
**Subcortical_L**	4	4	0	0	1	0	0	1	7	6	3	3	3	6	0	3
**Subcortical_R**	3	5	0	2	1	0	1	2	1	4	2	2	2	1	3	1

According to Supplementary Table 4, MC features that are predictive of pain sensitivity score were mainly in the following pairs of lobes (which have top 5 largest number of selected MC features): Prefrontal_L-Prefrontal_R, Temporal_L-Temporal_R, Prefrontal_L-Parietal_L, Prefrontal_L-Temporal_R, Prefrontal_L-Occipital_R, Prefrontal_R-Temporal_L.

3. Correlation between MC and GMV features

Supplementary Figure 2 shows the result of Pearson's correlation analysis between MC and GMV features. We can see that, frontal-subcortical MCs are significantly related to GMV of almost all regions.

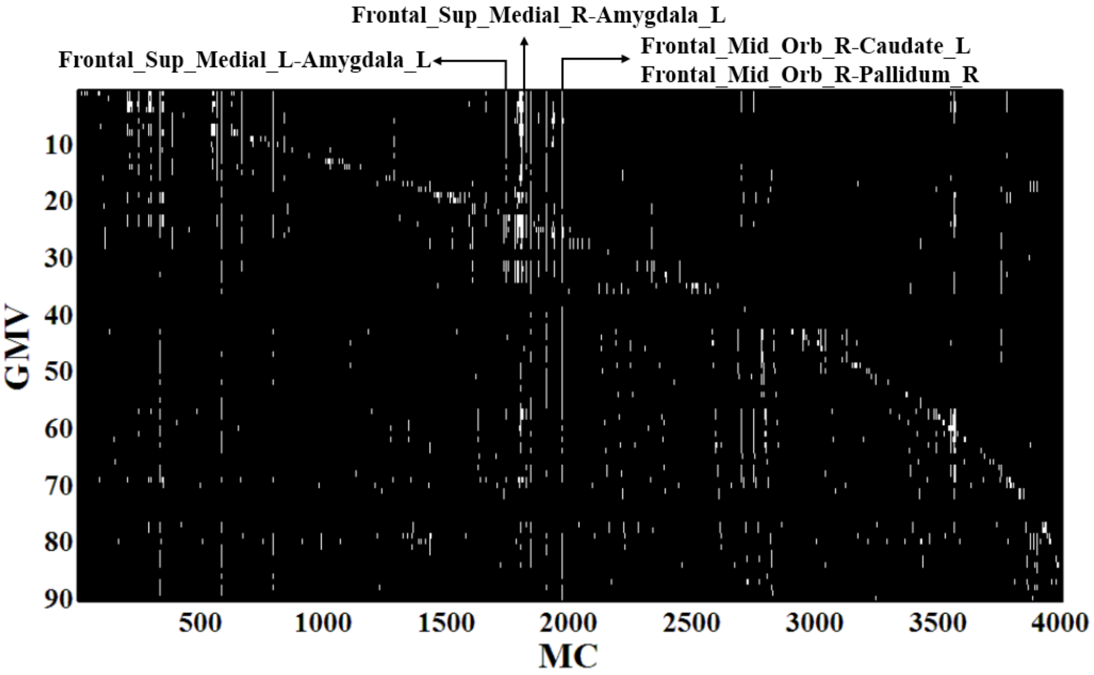

Supplementary Figure 2. Correlation analysis between MC and GMV features. A white point means a significant correlation ($P<0.05$, Bonferroni-corrected) between the corresponding MC and GMV. Some typical MCs that are correlated with GMV of almost all brain regions are labeled.

4. Comparison between PLSR and SVR

The regression model used in this study to predict pain thresholds is based on the PLSR. We also used another popular regression model, support vector regression (SVR), to predict pain thresholds. Supplementary Table 5 shows the prediction performance in predicting pain thresholds using PLSR and SVR. Supplementary Figure 3 shows the predictive MC features selected in PLSR and SVR.

Supplementary Table 5. Prediction performance in predicting three types of pain thresholds using three feature sets and two regression models (PLSR and SVR)

Pain threshold	Feature set	Regression model	MAE (mean±std)	MRAE (mean±std)	Correlation Coefficient
Laser pain threshold	MC	PLSR	0.28±0.23	0.11±0.11	0.73
		SVR	0.26±0.19	0.11±0.09	0.81
	GMV	PLSR	0.40±0.32	0.16±0.15	0.32
		SVR	0.41±0.33	0.16±0.15	0.17
	MC+GMV	PLSR	0.28±0.22	0.11±0.10	0.75
		SVR	0.25±0.19	0.10±0.09	0.81
Cold pain threshold	MC	PLSR	0.38±0.30	0.25±0.30	0.78
		SVR	0.34±0.22	0.23±0.23	0.85
	GMV	PLSR	0.62±0.46	0.42±0.48	0.24
		SVR	0.61±0.47	0.42±0.51	0.12
	MC+GMV	PLSR	0.37±0.28	0.24±0.26	0.80
		SVR	0.32±0.21	0.21±0.21	0.88
Pain sensitivity score	MC	PLSR	0.08±0.06	0.23±0.25	0.75
		SVR	0.09±0.05	0.28±0.26	0.70
	GMV	PLSR	0.11±0.09	0.33±0.41	0.38
		SVR	0.11±0.09	0.35±0.51	0.38
	MC+GMV	PLSR	0.07±0.05	0.22±0.25	0.77
		SVR	0.09±0.05	0.28±0.29	0.71

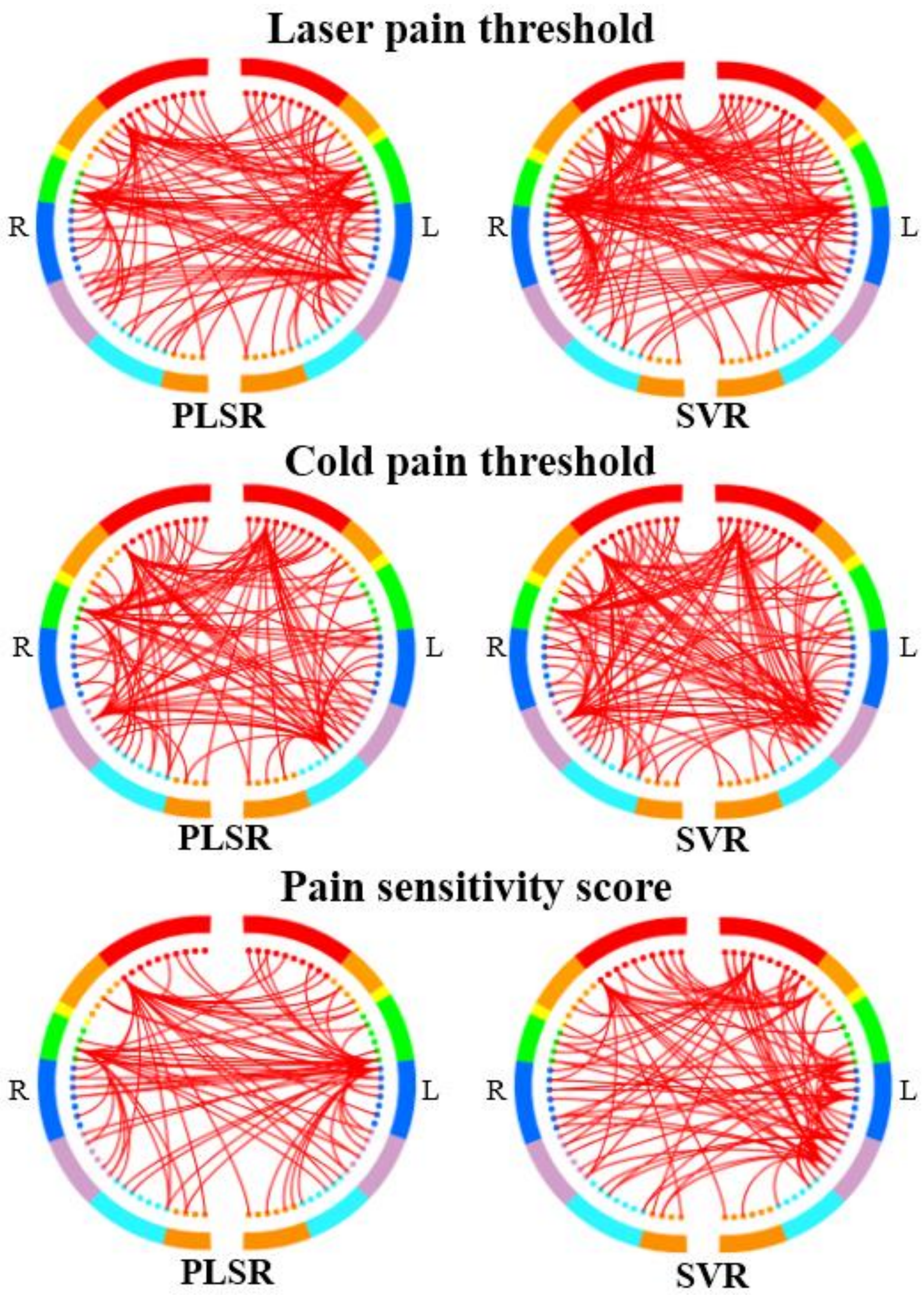

Supplementary Figure 3. Selected MC features for the prediction of different measures of pain sensitivity using two regression models (PLSR and SVR). The two half circles represent two brain hemispheres and the red lines represent the MC between two corresponding regions.
